# Relationship between ostomy caregiver skill proficiency and one-month risk of ostomy complications in patients with colorectal cancer: A cohort study

**DOI:** 10.1371/journal.pone.0355620

**Published:** 2026-08-12

**Authors:** Cui Lin, Fengying Zhao, Hui Chen, Feiyu Wang, Liquan Yang, Yifang Fang

**Affiliations:** 1 Department of Colorectal Surgery, The First Affiliated Hospital of Fujian Medical University, Fuzhou, Fujian, China; 2 Department of Endocrinology, The First Affiliated Hospital of Fujian Medical University, Fuzhou, Fujian, China; 3 School of Nursing, Fujian Medical University, Fuzhou, Fujian, China; 4 National Regional Medical Center, Binhai Campus of The First Affiliated Hospital, Fujian Medical University, Fuzhou, Fujian, China; 5 School of Nursing, Putian University, Putian, Fujian, China; Shuguang Hospital, CHINA

## Abstract

**Background**

Stoma-related complications often occur after ostomy, with an incidence of 21–70%. Clinical nursing mainly focuses on improving ostomy caregiver skill proficiency to reduce the occurrence of complications, but the specific relationship between caregiver competency proficiency and the incidence of stomatal complications is often overlooked. This study aims to evaluate the current status of ostomy caregiver skill proficiency among the caregivers of rectal cancer patients after surgery and to examine how these skills correlate with the risk of ostomy complications within the first month after surgery.

**Methods:**

This prospective study enrolled patients who underwent rectal cancer surgery with an ostomy between January 2022 and April 2024 and had no ostomy complications at discharge, together with their designated primary ostomy caregivers. Minimum discharge standards for home care of new ostomy patients were used as the evaluation tool to evaluate ostomy caregiver skill proficiency. The incidence of ostomy complications was followed up one month after surgery. Analysis of variance (ANOVA), the Cochran-Armitage test, and restricted cubic spline were used for statistical analysis.

**Results:**

Of 200 recruited patients and caregivers, 188 (94.0%) were included in the analysis. Ostomy caregiver skill proficiency was evaluated using WOCN consensus-based discharge criteria (range: 7–25), classifying 29 patients (15.4%) as low-skill (≤15), 123 (65.4%) as medium-skill (16–20), and 36 (19.1%) as high-skill (>20). During the first month post-discharge, 66 patients (35.1%) developed ostomy complications, defined as peristomal skin irritation, leakage, pouch-related issues, stoma retraction, or prolapse. Complication rates decreased with higher caregiver skill proficiency levels (75.9%, 30.1%, 19.4%; p < 0.001). Both the high-skill (adjusted odds ratio: 0.082) and medium-proficiency (adjusted odds ratio: 0.135) groups exhibited significantly low risk of complications. A restricted cubic spline curve further confirmed a significant nonlinear dose-response relationship between ostomy caregiver skill proficiency and the risk of ostomy complications in the first month after surgery (p= 0.021). Receiver operating characteristic curve analysis showed an area under the curve of 0.719 (95% CI:0.637–0.800), indicating **moderate predictive ability**.

**Conclusion:**

Higher levels of ostomy caregiver skill proficiency were associated with a substantially lower risk of early postoperative ostomy complications. Although the predictive performance was moderate, caregiver skill assessment may provide useful information for early risk identification and targeted intervention. These findings highlight the importance of strengthening caregiver training to improve early postoperative outcomes and suggest a potential skill threshold that may help clinicians identify patients at higher risk. Further validation in larger, multicenter studies is warranted.

## Introduction

Colorectal cancer is a common malignant tumor of the gastrointestinal tract. With its increasing incidence and mortality, the number of patients requiring ostomy surgery is also increasing. Ostomy surgery involves creating an abdominal opening to divert feces from the colon or rectum into an external bag, serving as an essential intervention for managing colorectal diseases [1]. However, this procedure is associated with complications such as peristomal dermatitis. Inadequate postoperative care can significantly impact a patient’s quality of life [2]. Therefore, ensuring optimal ostomy care is crucial for preventing complications and promoting recovery.

Ostomy care involves proper cleaning and management of the stoma to prevent infection and promote healing, playing a crucial role in improving the quality of life for ostomy patients [3]. Effective care requires skilled techniques, appropriate ostomy system selection, proper baseplate use, and adequate skin protection. Studies have shown that inadequate caregiver skills, such as improper pouch changes and inaccurate skin assessments, are linked to higher rates of skin irritant dermatitis (up to 57%), leakage (30–40%), and an increased risk of infection [4]. A study found that inadequate care techniques significantly increase complications, underscoring the need for improved training programs. Systematic reviews [5] indicate that involvement of professional ostomy nurses reduces complication risk by 42%, particularly during the critical 30-day postoperative period. These findings emphasize the importance of professional support in minimizing adverse outcomes, yet gaps remain in the implementation of such support. A randomized controlled trial demonstrated that standardized training programs reduced nursing-related complications by 35% [6]. Furthermore, longitudinal studies indicate that only 46% of patients adhere to self-care practices within 3 months after discharge. A study [7] found that inadequate self-care after discharge directly increases the risk of complications. Ostomy caregiver skill proficiency is increasingly recognized as a key factor affecting the occurrence of ostomy complications. However, research on the relationship between proficiency in ostomy care and postoperative complications remains limited. This study aims to assess the current status of ostomy caregiver skill proficiency and its association with ostomy-related complications in rectal cancer patients one month after surgery, providing valuable insight to reduce the risk of early postoperative complications. We hypothesized that higher ostomy caregiver skill levels at discharge would be associated with a lower risk of early ostomy complications.

## Methods

### Study design and participants

The prospective study included patients who underwent surgery for rectal cancer between January 2022 and April 2024 at a tertiary hospital in Fujian Province, China. The patients had no ostomy complications at discharge and had a designated primary ostomy caregiver. The inclusion criteria were: (1) pathologically confirmed rectal adenocarcinoma, (2) undergoing ostomy surgery, (3) no ostomy complications at discharge, (4) the presence of a designated primary ostomy caregiver, and (5) individuals with strong hands-on skills, unobstructed vision, and the ability to provide proper care. The exclusion criteria were: (1) patients who were unwilling to participate, (2) patients who were unwilling to cooperate with follow-up, (3) those with cognitive impairment or psychiatric disorders preventing communication or reliable data collection, and (4) the inability to identify a primary ostomy caregiver.

A total of 200 ostomy patients and caregivers were recruited. The sample size was estimated using a single-proportion formula:


$$\begin{array}{c} n=\frac{Z_{\alpha /\text{2}}^{\text{2}}\times p\times (\text{1}-p)}{d^{\text{2}}} \end{array}$$

Reported [8] rates of peristomal complications range from 20% to 70% in the literature. For the purpose of calculation, we assumed an expected incidence of 40% (p = 0.40). With α = 0.05 (two-sided), β = 0.20 (power = 80%), and a margin of error of 0.07, the required sample size was calculated. To account for potential loss to follow-up, we increased the target sample size by approximately 10%, resulting in an intended enrollment of about 200 participants. In addition, we considered the requirements of multivariable logistic regression for adjusted analyses. According to the rule of at least 10 outcome events per predictor variable, an expected event rate of 40% would yield sufficient events to include three to four covariates in the final model without risk of overfitting. Recruitment feasibility and data completeness were closely monitored throughout the study period. The actual achieved sample size, along with any losses to follow-up, is reported in the Results section.

In this study, the ostomy caregiver skill proficiency of patients at discharge was assessed using the Wound, Ostomy, and Continence Nursing (WOCN) Consensus Conference’s minimum discharge standards for home care of new ostomy patients as the evaluation tool [9] in 2016. The criteria were established through expert consensus and content validation, but no published studies have reported formal psychometric testing of the tool.

During implementation in our study, two checklist items (items 12 and 14) contained multiple procedural components that were difficult to score consistently in routine clinical assessment. Item 12 was subdivided into five operational sub-items and item14 into four operational sub-items to improve scoring clarity and standardize evaluation. These modifications involved only the scoring format and item numbering. No assessment content, competency domains, or scoring criteria were changed, and the original construct measured by the WOCN checklist was preserved. Caregiver skill proficiency was categorized into three groups according to the total assessment score for analytical purposes: low proficiency (<15 points), medium proficiency (15–20 points), and high proficiency (>20 points). These categories were created to facilitate comparison among different levels of caregiver competency based on the observed score distribution and clinical interpretability, rather than to define validated diagnostic thresholds.

The cross-sectional survey data on ostomy caregiver skills at discharge were used as the independent variable, with these groups serving as the exposure factors in this cohort study. The outcome measure was the presence or absence of ostomy complications in the first month after surgery.

This study was conducted in accordance with the principles of the Declaration of Helsinki and was approved by the Ethics Committee of the First Affiliated Hospital of Fujian Medical University (approval number: MRCTA, ECFAH of FMU [2021] 187; MRCTA, ECFAH of FMU [2022] 363). Written informed consent was obtained from both patients and their caregivers before enrollment in the study. Participants were fully informed about the study objectives, procedures, potential risks, and benefits. They were assured that their data would remain confidential and anonymous. Participation was voluntary, and participants were informed of their right to withdraw from the study at any time without any impact on their medical care.

### Variable and measures

The assessment tool used in this study to assess ostomy caregiver skill proficiency was adapted from the Minimum Discharge Standards for Home Care of New Ostomy Patients, as outlined by the WOCN Consensus Conference [10]. Initially consisting of 18 items, the tool was expanded and localized to 25 items for this study. The content covered areas such as identifying normal and abnormal ostomy appearance, emptying the ostomy bag, and managing complications. Each item was scored with 1 point for mastery and 0 points for nonmastery or lack of knowledge, with a maximum score of 25 points.

At discharge, a clinical expert in ostomy care communicated with both the patient and the caregiver, and after obtaining their consent, the patient was included in the study. The caregiver’s proficiency in ostomy care skills was comprehensively assessed and recorded. The patient’s ostomy was evaluated for the presence or absence of ostomy-related complications. Comprehensive data were collected, including age, height, gender, weight, ostomy height, and the length and width of the ostomy. The first postoperative month was chosen as the observation window because most ostomy-related complications, including peristomal skin irritation, leakage, and appliance-related issues, typically occur shortly after discharge when patients or caregivers begin home management. By focusing on this period, the study captures complications most directly influenced by discharge competency. During this period, due to the pain from the surgical wound, the patient’s psychological resistance to the stoma was high, and the patient’s dependence on the family or caregivers was also significant. As a result, most patients were unwilling to change the stoma themselves. Age and body mass index (BMI) are important factors in predicting surgical outcomes and complications related to the stoma. Compared with younger patients, older patients tend to recover more slowly, have a heavier burden of complications, and experience more complications. Studies [11] have demonstrated that age, BMI, and stoma size should be included as important covariates. Gender [12], although it is an important variable in many clinical outcomes, has not been found to be a significant factor for stoma complications when other key clinical variables are taken into account.

The primary outcome of this study was the presence or absence of ostomy complications within the first month after surgery. One month after discharge, follow-up assessments were conducted by a clinical specialist in ostomy nursing through outpatient visits and WeChat video calls to monitor the occurrence of ostomy complications, including peristomal dermatitis (follow-up rate = 94%).

The anonymized participant-level dataset is provided as S1 Dataset.

### Statistical analysis

All data were processed using R software (version 4.2.2) and MSTATA software (www.mstata.com). Continuous variables are expressed as means ± standard deviations (SD), while categorical variables are reported as frequencies (proportions). Descriptive statistics were used to summarize participants’ baseline characteristics and ostomy care skill scores. Analysis of variance (ANOVA) and the Cochran-Armitage test were used to compare baseline characteristics among the three skill level groups and examine trends in ostomy complications, assessing the linear relationship between skill levels and complication incidence.

Sample size estimation was performed based on the requirements for the planned multivariable logistic regression analysis to ensure an adequate number of outcome events for model development and adjustment for potential confounders. Based on the expected complication rate of approximately 40% and the commonly recommended minimum number of outcome events per predictor variable, the available sample size was considered sufficient for the primary multivariable model while minimizing the risk of overfitting.

Because the number of potential confounding variables exceeded the number that could reasonably be accommodated in the primary model, least absolute shrinkage and selection operator (LASSO) regression with 10-fold cross-validation was first performed to reduce dimensionality and identify the most informative variables. Variables retained by LASSO were subsequently entered into the primary multivariable logistic regression model using the Enter method. In addition, a second prespecified robustness model was constructed by further including several clinically relevant covariates (age, BMI, ostomy height, ostomy length, ostomy width, and distance from the stoma to the abdominal midline) that were not retained by LASSO but were considered clinically important. This second model was intended as a sensitivity analysis to evaluate the robustness of the observed associations rather than as the primary inferential model.

Before logistic regression analyses, model assumptions were assessed. Multicollinearity was evaluated using variance inflation factors (VIF), with VIF values <5 considered acceptable. Approximate linearity between continuous covariates and the logit of the outcome was examined. Observation independence was ensured by the prospective cohort design, with each participant contributing only one observation. Model convergence was confirmed, and influential observations and extreme outliers were examined before final model fitting.

Restricted cubic spline (RCS) analysis was conducted to explore the dose-response relationship between ostomy caregiver skill proficiency and the risk of ostomy complications one month after surgery. Receiver operating characteristic (ROC) curve analysis was used to assess the predictive value of ostomy caregiver skill proficiency for complication risk within the first month after surgery, as well as to identify the critical value for ostomy caregiver skills. All tests were two-tailed, with statistical significance set at p < 0.05.

Potential confounders were selected from prospectively collected baseline demographic and clinical variables that were available in the study database. Several clinically relevant factors, including patient comorbidities, nutritional status, caregiver educational level, caregiver health literacy, previous caregiving experience, and post-discharge support, were not routinely collected and therefore could not be included in the present analyses.

## Results

### Baseline characteristics and ostomy caregiver skills proficiency

**Fig 1:**
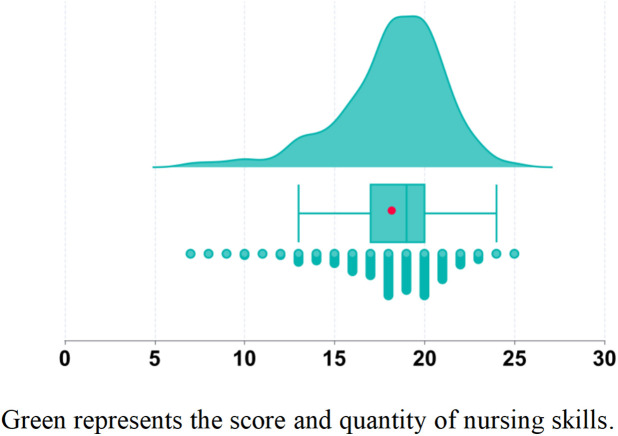
Distribution of caregiver skill scores. Green represents the score and quantity of ostomy caregiver skill proficiency.

A total of 200 patients were recruited, providing 188 valid responses (94.0%). Nine patients (4.5%) were excluded due to incomplete data, and three patients (1.5%) were lost to follow-up. Therefore, the final analytic sample consisted of 188 participants. The ostomy caregiver skill scores of the patients with rectal cancer at discharge ranged from 7.00 to 25.00, with a mean score of 18.17 ± 2.97 (Fig 1). Patients were stratified into three groups according to their caregivers’ostomy care skill proficiency (high, medium, low). Among the participants, 29 patients (15.43%) were in the low-skill group, 123 patients (65.42%) were in the medium-skill group, and 36 patients (19.15%) were in the high-skill group. Compared to the high-skill group, patients in the low-skill group had a significantly higher BMI (p < 0.05). Baseline demographic and clinical characteristics of the study participants are summarized in Table 1. Compared with the high-proficiency group, patients in the low-proficiency group had a significantly higher BMI and larger abdominal circumference, whereas no significant differences were observed among the groups in terms of ostomy height or dimensions (length, width) (p > 0.05).

**Table 1:** Patient demographics and baseline characteristics.

Characteristic	low, N = 29	medium, N = 123	high, N = 36	F/*χ*^*2*^	p-value
age	60.83 ± 13.02	62.01 ± 11.63	61.97 ± 11.94	0.119	0.888
BMI	24.27 ± 4.33	23.63 ± 3.54	22.12 ± 2.93	3.421	0.035
Employment status				0.111	0.946
Unemployed	16(55.2)	72(58.5)	21(58.3)		
Employed	13(44.8)	51(41.5)	15(41.7)		
Visual acuity status				1.404	0.512
Normal vision	16(55.2)	77(62.6)	25(69.4)		
Myopia/Hyperopia	13(44.8)	46(37.6)	11(30.6)		
Manual dexterity	29(100)	123(100)	36(100)	–	>0.999
Abdominal circumference	85.31 ± 5.17	83.15 ± 4.34	81.89 ± 4.94	4.529	0.012
Abdominal skin condition				–	>0.999
Intact	27(93.1)	115(93.5)	34(94.4)		
Scarred	2(6.9)	8(5.7)	2(5.6)		
self-care ability				0.929	0.629
completely	26(89.7)	116(94.3)	33(91.7)		
Notcompletely	3(10.3)	7(5.7)	3(8.3)		
Type of ostomy				–	0.030
End ostomy	0(0.0)	2(1.6)	4(11.1)		
Loop ostomy	29(100.0)	121(98.4)	32(88.9)		
Distance from stoma to umbilicus (cm)	6.45 ± 0.82	6.73 ± 0.94	6.78 ± 0.77	1.365	0.258
Distance from stoma to abdominal midline (cm)	3.48 ± 0.71	3.62 ± 0.70	3.79 ± 0.67	1.641	0.197
height	1.77 ± 0.40	1.69 ± 0.33	1.77 ± 0.42	0.505	0.6043
length	4.08 ± 0.48	4.30 ± 0.42	4.26 ± 0.61	1.707	0.1843
width	2.08 ± 0.31	2.13 ± 0.32	2.14 ± 0.33	0.398	0.6723
Color of stoma				–	0.664
beef-colored	1(3.4)	1(0.8)	0(0.0)		
dark red	4(13.8)	15(12.2)	4(11.1)		
edematous appearance	24(82.8)	107(87.0)	32(88.9)		
Peristalsis of stoma				1.764	0.414
Normal	13(44.8)	64(52.0)	22(61.1)		
slowly	16(55.2)	59(48.0)	14(38.9)		
Peristomal abdominal contour				–	0.021
Retracted	0(0.0)	0(0.0)	3(8.3)		
Protuberant	0(0.0)	6(4.9)	2(5.6)		
Flat	29(100)	117(95.1)	31(86.1)		
Peristomal abdominal wall consistency				–	0.746
Firm	1(3.4)	4(3.3)	1(2.8)		
Soft	16(55.2)	69(56.1)	19(44.4)		
Mucocutaneous junction integrity				–	>0.999
Intact	29(100.0)	123(100.0)	36(100.0)		
Non-intact	0(0.0)	0(0.0)	0(0.0)		
Ostomy caregiver skill proficiency group				26.383	<0.0014
no	29 (80.6%)	7 (24.1%)	86 (69.9%)		
yes	7 (19.4%)	22 (75.9%)	37 (30.1%)		

Patient demographics and clinical characteristics are presented according to caregiver skill proficiency groups (low, medium, and high). Continuous variables are expressed as mean ± standard deviation (SD), and categorical variables as number (percentage). Differences between groups were assessed using appropriate statistical tests.

### Prevalence of complications

**Fig 2:**
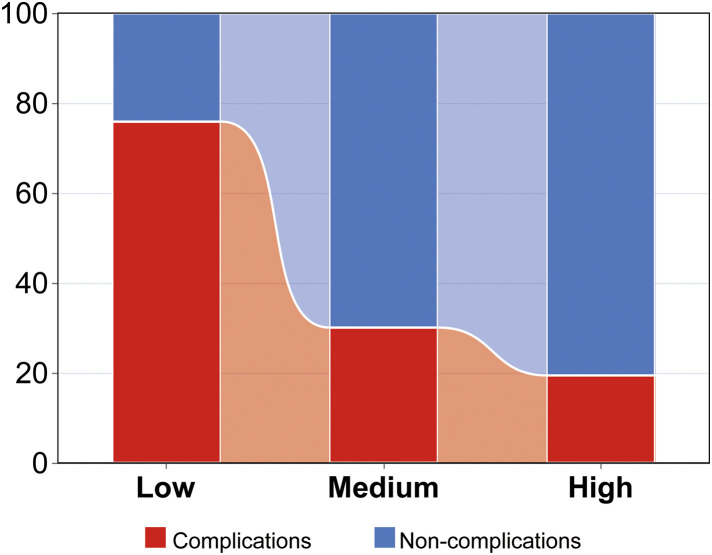
Complication rate by caregiver skill group. One-month risk rate of stoma complications in patients with low, medium, and high stoma care skills.

**Fig 3:**
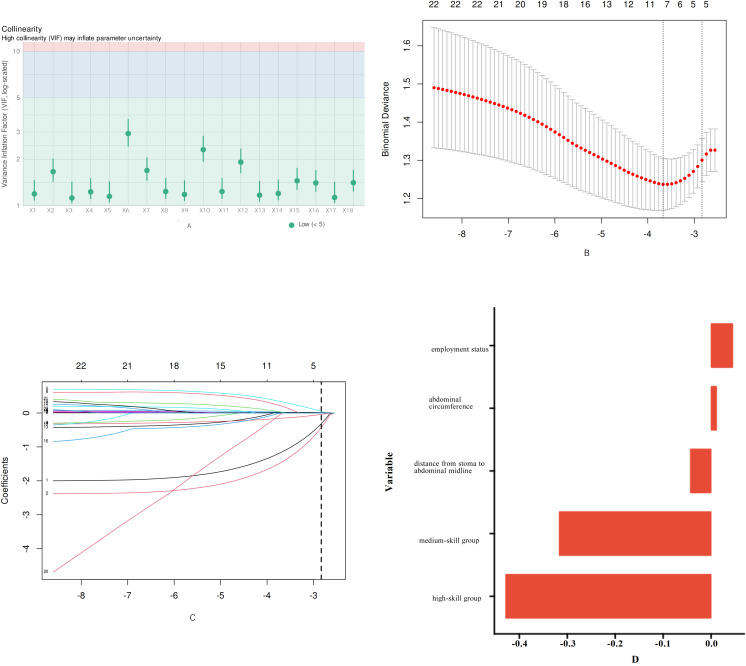
**Variable selection and collinearity diagnostics for the multivariable logistic regression analysis.** (A) Collinearity diagnostics of candidate covariates. (B) Ten-fold cross-validation for LASSO model selection. (C) LASSO coefficient profiles across penalty parameters. (D) Variables selected by LASSO at the optimal penalty parameter.

**Fig 4:**
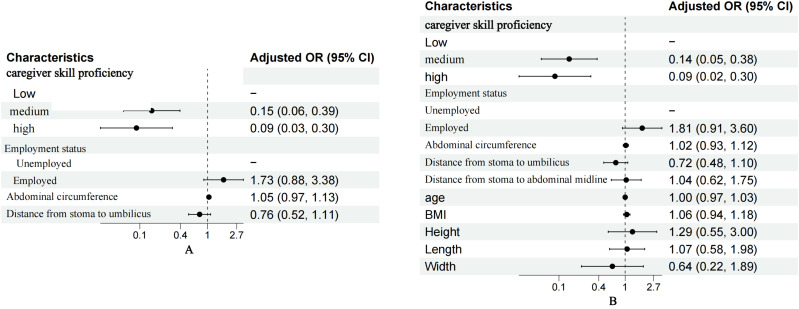
Univariate analysis of influencing factors (forest map). Odds ratio (OR; 95% confidence interval (CI)) represents the result of univariate logistic regression analysis, while adjusted OR (95% CI) represents the result of multivariate logistic regression analysis. A. Primary multivariable logistic regression model based on LASSO-selected variables. B. Robustness multivariable logistic regression model with additional clinically relevant covariate adjustment.

At the one-month postoperative follow-up,66 patients (35.11%) had developed ostomy complications during the first month after rectal cancer ostomy surgery. Ostomy complications were defined as any of the following occurring from hospital discharge to one month postoperatively: peristomal skin irritation, leakage, pouch-related issues, stoma retraction, or stoma prolapse. As shown in Fig 2, the patients were categorized into low-, medium-, and high-skill caregiver groups based on WOCN consensus-derived discharge criteria, with low-skill defined as ≤15 points, medium-skill as 16–20 points, and high-skill as >20 points. The high ostomy care skill groups were 75.86%, 30.08%, and 19.44%, respectively. The Cochran-Armitage test revealed a significant linear trend between these variables (p < 0.001). Logistic regression analysis revealed a significant association between ostomy caregiver skill proficiency and the risk of ostomy complications at one month (Fig 3A-3D). Compared to the low-skill group, the high-skill group (adjusted odds ratio (OR), 0.082; 95% CI, 0.024–0.274; p < 0.001) and the medium-skill group (adjusted OR, 0.135; 95% CI, 0.053–0.348; p < 0.001) had significantly lower risks of ostomy complications at one month (Fig 4A). Additionally, age, BMI, ostomy height, ostomy length, and ostomy width were not significantly associated with the risk of complications, with adjusted ORs of 1.001 (95% CI, 0.973–1.030; p = 0.934), 1.058 (95% CI, 0.965–1.160; p = 0.229), 1.290 (95% CI, 0.564–2.947; p = 0.546), 1.046 (95% CI, 0.573–1.911; p = 0.883), and 0.773 (95% CI, 0.275–2.172; p = 0.625), respectively (Fig 4B).

### Dose-response relationship between ostomy caregiver skill proficiency and the one-month risk of ostomy complications

**Fig 5:**
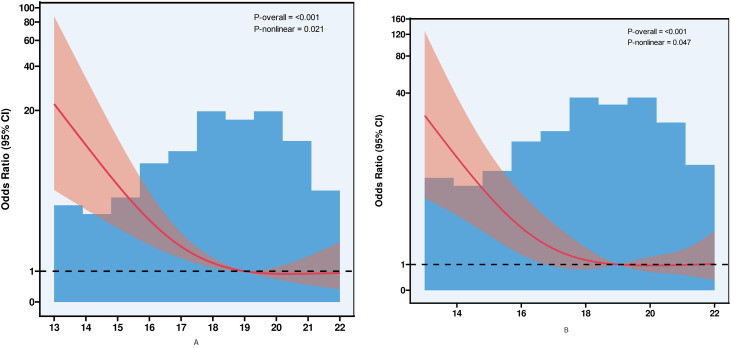
Association between ostomy caregiver skills and complications using the RCS function. Association between ostomy care skills and complications with the RCS function. Model with three knots located at the 10^th^, 50^th^, and 90^th^ percentiles. The Y-axis represents the OR to present a complication for any value of ostomy care skills compared to individuals with ostomy care skills within the reference value (50^th^ percentile). A. Unadjusted restricted cubic spline model. B. Adjusted restricted cubic spline model.

The X-axis in Fig 5 represents the level of ostomy caregiver skills, while the Y-axis represents OR for the risk of ostomy complications, along with the corresponding 95% confidence interval (CI). The RCS curve demonstrated the relationship between ostomy caregiver skill proficiency and the risk of ostomy complications from hospital discharge to one month postoperatively. A significant nonlinear dose-response relationship was observed in the unadjusted model (test for nonlinearity, p = 0.021). However, the reduction in complication risk plateaued at higher skill levels (Fig 5A). After adjusting for covariates such as age, BMI, ostomy height, ostomy length, and ostomy width, this nonlinear relationship remained statistically significant (test for nonlinearity, p = 0.018), as shown in Fig 5B.

### ROC curve analysis of ostomy caregiver skill proficiency predicting the risk of ostomy complications

**Fig 6:**
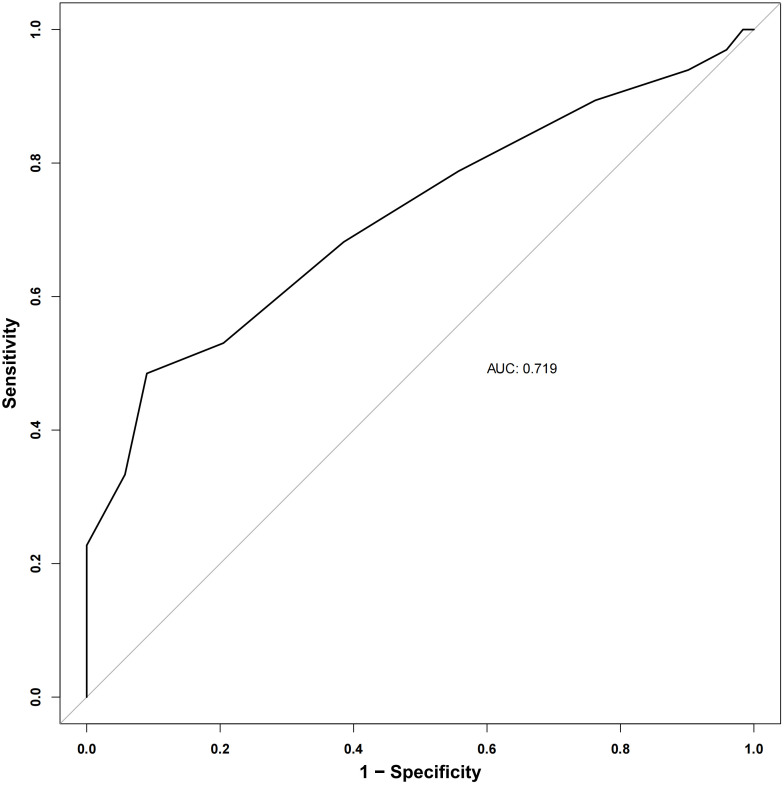
Receiver operating characteristic (ROC) curve evaluating the ability of ostomy caregiver skill proficiency to discriminate the risk of one-month ostomy complications.

Fig 6 shows that the area under the curve for assessing the risk of ostomy complications one month after surgery based on ostomy caregiver skill proficiency is 0.719 (95% CI: 0.637–0.800),indicating **moderate discriminatory performance**. The optimal threshold for predicting complications was established using the maximum Youden index, which reached 0.395. At this threshold, the sensitivity was 0.910, while the specificity was 0.485, corresponding to a caregiver skill proficiency score of 16.5. Although the sensitivity was relatively high, the specificity was limited, suggesting that the predictive performance of caregiver skill proficiency alone remains moderate. Nevertheless, it may provide useful information for identifying patients at increased risk of ostomy complications, particularly in the early postoperative period.

## Discussion

The study findings revealed a significant linear relationship between ostomy caregiver skill proficiency at discharge and the risk of ostomy complications within the first month after surgery. Patients in the high- and medium-skill groups demonstrated significantly lower odds of ostomy complications compared to those in the low-skill group. The linear trend suggests an inverse association between ostomy caregiver skill proficiency and ostomy complications [13,14]. Importantly, our RCS analysis revealed a nonlinear dose-response relationship, indicating that the association between caregiver skill proficiency and complication risk plateaued beyond a certain proficiency threshold. This suggests that while foundational skills were associated with fewer early complications, additional incremental improvements beyond a moderate-high skill level may confer diminishing returns. Clinically, this emphasizes the need to ensure that all caregivers reach a minimum competency level rather than solely focusing on incremental mastery in already proficient individuals.

The results of this study align with previous research, further indicating an association between ostomy caregiver skill proficiency and the risk of complications following ostomy surgery, consistent with the findings of Stenberg, which revealed that patients with better ostomy care knowledge experienced fewer complications. [13,15,16] Key ostomy caregiver skill proficiency **that may be associated with** lower complication rates include stoma care, appliance management, peristomal skin assessment, and patient education [17]. Our study findings also suggest that **higher** ostomy caregiver skill proficiency **is associated with** lower odds of ostomy complications [18–20]. Gupta reported [21–23] that effective ostomy care education and training are essential in preventing peristomal complications, a notion [24] that is **consistent with the association observed in our study**, in which patients with higher ostomy caregiver skill proficiency exhibited a significantly lower incidence of complications.

In routine clinical practice, nursing staff often prioritize teaching patients or caregivers the basic skills needed to manage their ostomies independently at home, typically focusing on technological processes such as how to change the ostomy appliance. However, they may not consider the impact of skill mastery on the occurrence of complications, given the concept of rapid medical and surgical rehabilitation and limited hospital stays.

A unique contribution of our study is the identification of a nonlinear dose-response relationship between ostomy caregiver skill proficiency and the occurrence of ostomy complications by **quantifying this nonlinear association and identifying a practical threshold for skill proficiency.** This finding provides valuable insight into the effect of ostomy caregiver skill proficiency on postoperative ostomy complication outcomes. **Stenberg** [25] found that, beyond a moderate level of proficiency, additional gains in caregiver skill may yield smaller improvements. **Our findings suggest that caregiver skill proficiency may serve as a useful marker for identifying patients at increased risk of postoperative complications.** Implementing competency-based training programs targeting caregivers below the identified threshold may help support early postoperative outcomes, improve patient safety, and guide the efficient allocation of educational resources.

In summary, this study identified an inverse association between ostomy caregiver skill proficiency and early postoperative complications and provides novel insight into the dose-response dynamics between skill level and complication risk. These findings suggest that structured, competency-based ostomy education and clear skill thresholds may be useful for discharge assessment and caregiver training in clinical practice. Future studies should evaluate the long-term benefits of digital or mHealth-assisted caregiver training.

### Limitations

Several limitations should be acknowledged. First, this cross-sectional survey design conducted at discharge limits our ability to establish causal relationships between ostomy caregiver skill proficiency and the risk of ostomy complications.

Second, the study employed only a one-month postoperative longitudinal follow-up, which may introduce bias due to the relatively short time frame.

Third, the sample was limited to a single hospital in Fujian Province, China, which may affect the generalizability of the findings to other regions or countries with different healthcare systems and patient populations.

Fourth, while ostomy care proficiency was assessed by professionals at discharge, we did not have the opportunity to observe the performance of patients or caregivers in their home environments [13,16,26].

Fifth, although we adjusted for a range of demographic and clinical characteristics and conducted additional robustness analyses, residual confounding cannot be completely excluded. Some clinically relevant and contextual factors, including patient comorbidities, nutritional status, smoking status, caregiver educational level, caregiver health literacy, previous caregiving experience, and the availability of post-discharge community support, were not routinely collected and therefore could not be included in the analyses. These unmeasured factors may have associations with both caregiver skill proficiency and postoperative outcomes. Accordingly, the observed associations should be interpreted with appropriate caution.

Future research should address these limitations by conducting multicenter longitudinal studies and including real-world assessments of ostomy care performance. Integrating caregiver competency with clinical and biological factors may further improve predictive performance.

## Conclusion

Higher ostomy caregiver skill proficiency was **associated with a lower likelihood** of ostomy complications during the first postoperative month after colorectal cancer surgery. The observed association remained stable after adjusting for measured confounding variables and sensitivity analyses. Given the observational nature of this study, these findings should not be interpreted as evidence of a causal relationship. Rather, they suggest that caregiver skill proficiency **may serve as a useful indicator** for identifying patients who could benefit from enhanced discharge education and postoperative support. Future multicenter prospective studies using validated assessment instruments are warranted to confirm these findings.

## Supporting information

S1 Dataset Anonymized participant-level dataset used in this study. Minimal data set supporting the findings of this study. This file contains the anonymized dataset used for all statistical analyses, including caregiver demographic information, ostomy care skill scores, complication outcomes, and related covariates. All personally identifiable information has been removed to protect participant confidentiality.
(XLSX)
